# Measuring the Effects of Social Isolation and Dissatisfaction on Depressive Symptoms during the COVID-19 Pandemic: The Moderating Role of Sleep and Physical Activity

**DOI:** 10.3390/brainsci11111449

**Published:** 2021-10-30

**Authors:** Ivan Vargas, Erin Kaye Howie, Alexandria Muench, Michael L. Perlis

**Affiliations:** 1 Department of Psychological Science, University of Arkansas, 310 Memorial Hall, Fayetteville, AR 72701, USA; 2 Department of Health, Human Performance and Recreation, University of Arkansas, Fayetteville, AR 72701, USA; ekhowie@uark.edu; 3 Department of Psychiatry, University of Pennsylvania, Philadelphia, PA 19104, USA; amuench@pennmedicine.upenn.edu (A.M.); mperlis@upenn.edu (M.L.P.)

**Keywords:** COVID-19, depression, social isolation, sleep, physical activity

## Abstract

Social distancing was universally implemented to reduce the spread of the COVID-19 virus. Long-term social distancing can lead to increased feelings of social isolation or dissatisfaction with one’s daily interpersonal interactions, which can subsequently result in reduced psychological health (e.g., greater depression). The present study quantified this association, and the extent to which it was moderated by measures of sleep and physical activity, by surveying 3658 adults (mean age = 46.0 years) from across the United States. Participants answered questions related to their social experiences, sleep, physical activity, and depressive symptoms during the early stages of the pandemic (March–June 2020). Results showed that social isolation and social dissatisfaction were associated with greater depressive symptoms. As predicted, self-reported sleep quality and physical activity moderated these associations, such that lower sleep quality and physical activity exacerbated their effect on depressive symptoms.

## 1. Introduction

The COVID-19 pandemic was declared a national emergency in the United States on 13 March 2020, with most states issuing stay-at-home orders during the subsequent weeks. The present pandemic has presented a unique situation in which people were almost universally engaging in the public health intervention known as social distancing. Social distancing aims to minimize the impact and transmission of infectious diseases by reducing the frequency and duration people spend in proximity with one another [1]. This public health strategy is critical for slowing the transmission of COVID-19 [2,3], however, there have been some unintended negative consequences. One consequence is the negative effect it has had on psychological health and mood symptoms, especially among COVID-19 patients [4]. While this concern was initially put forward by the World Health Organization [5], it has now been supported by multiple publications (see [6,7,8,9,10] for more information). Most of this research, however, has been conducted on healthcare workers, and so the overall impact on the general population is less clear. Moreover, the specific factors that have contributed to the decreases in overall psychological health remain vague or elusive. Two important and related social factors that are critical for psychological well-being are having a sense of community and the overall satisfaction in one’s interpersonal relationships [11,12]. Both have been significantly impacted during the COVID-19 pandemic, albeit in nuanced ways. While physical contact with friends and family that live separately has decreased, virtual contact with others has increased. Furthermore, while time spent with family members has gone up, physical contact with peers, colleagues, and friends has gone down. Overall, these changes have likely led to unmet social needs (i.e., social isolation and/or dissatisfaction with one’s daily social interactions) during the past year and a half [13].

A presumed mechanism by which social distancing negatively affects mood and/or psychological well-being is social isolation. The effects of social isolation on psychological health have been relatively well-documented in non-human animals, with several studies showing that following extended periods of social isolation, animals are more likely to demonstrate depressogenic impairments in behavior [14,15]. There is also some research documenting the association between social isolation and psychological health in humans, however, these studies are often limited to special populations (e.g., prisoners) [16,17], and therefore, it is unclear whether these findings will generalize to others. The relative significance of social distancing is also supported by studies showing that a lack of social connectedness or support is related to greater psychopathology, including depression [11,12]. Low social connectedness or high social isolation have even been identified as significant risk factors for suicidality (i.e., see Interpersonal Theory of Suicide) [18,19]. Given these considerations, the first aim of the present study was to assess whether greater social isolation and/or overall dissatisfaction with one’s social interactions were related to worse psychological health (i.e., greater depressive symptoms) in the United States during the early stages of the COVID-19 pandemic (i.e., March to June 2020).

It may be the case that social isolation or social dissatisfaction are associated with greater depressive symptoms, especially during a global pandemic. That said, it is also important to consider that there are several factors that can simultaneously impact the frequency or severity of a person’s depressive symptomatology. That is, because the social consequences of the pandemic are unavoidable (i.e., stay-at-home orders are inevitably going to lead to increased feelings of social isolation or dissatisfaction for many), it is critical to identify what other factors may buffer a person’s psychological health during this time. Two such factors with well-documented links to depression are sleep and physical activity [20,21,22]. Poor sleep, and in particular insomnia, is a known risk factor for depression [23,24]. Similarly, individuals who are more physically active are less likely to report symptoms of depression [25,26,27]. Interventions focused on improving sleep and physical activity have been shown to reduce depressive symptomatology [28,29,30]. The potential effects of sleep and physical activity are even more relevant given that data supports a shift in sleep patterns and increases in insomnia symptoms among people, especially healthcare workers, that were surveyed during the COVID-19 pandemic [8,31,32,33,34]. People have also become significantly less active during the pandemic [35,36,37]. Lower physical activity and greater sleep loss have been linked to greater loneliness and social isolation [13,38,39,40], and therefore, it is possible that getting good sleep or being physically active may be protective against the effects of social isolation or dissatisfaction. For example, individuals who are experiencing high levels of social isolation may experience less depression if their sleep quality and/or physical activity are high. Therefore, the second aim of the present study was to assess whether sleep or physical activity moderated the relationships between social isolation and dissatisfaction and depressive symptoms.

## 2. Materials and Methods

### 2.1. Participants and Procedure

The original sample included 4052 adults (3184 cisgender females, mean age = 46.0 years) recruited from the United States to participate in an online study on social distancing and mood. Study recruitment occurred between March and June 2020 through postings on multiple social media websites (e.g., Facebook, Reddit), an email list of former research subjects that had previously completed a sleep screener, online newsletters, and ResesarchMatch. Prospective participants were included in the study if they were at least 18 years old, had access to the Internet, and were able to read and write in English. Eligible individuals were first invited to complete a baseline survey followed by twice daily brief surveys (one in the morning and one in the evening) for two weeks. These surveys assessed social distancing, mood, sleep, physical activity, and basic demographic information (see Measures section below). Participants were instructed to complete the morning (AM) survey after waking up and the evening (PM) survey before going to bed. The surveys were administered via Qualtrics XM (Provo, UT, USA). Participants were included in the subsequent analyses if they completed at least one of the daily assessments (range = 1–14 days). Of these, 394 participants were excluded from the final analyses since they only completed the baseline survey (i.e., no daily assessments). The final sample included 3658 participants (2885 cisgender females, mean age = 45.9 years) and did not significantly differ in age, gender, or race compared to those that were excluded. The final sample included 83.2% white or Caucasian, 4.8% black, African American or African, 3.2% Latino or Latina, 4.1% Asian or Asian American, 0.5% American Indian, Native American, or Alaska Native, 0.3% Middle Eastern or Arab, 0.1% Native Hawaiian or other Pacific Islander, 2.8% Multi-racial, and 0.9% who self-identified as “Other”. Approximately 70% of the final sample completed at least half or seven of the 14 daily assessments, and more than 75% of the sample completed at least five daily assessments. See Table 1 for a demographic summary of the final sample.

### 2.2. Measures

#### 2.2.1. Social Isolation and Satisfaction

Social isolation and social satisfaction were assessed using one-item Likert-based measures. For social isolation, participants were asked to rate on a 0 to 100 scale “to what extent you felt socially isolated TODAY?” (0 = “Not socially isolated at all” and 100 = “Very socially isolated”). For social satisfaction, participants were asked to rate “how satisfied are you with the quality and quantity of your social interactions today?”. This was measured on a 7-point Likert scale from “extremely dissatisfied” (1) to “extremely satisfied” (7). These questions were included as part of the daily PM survey.

#### 2.2.2. Sleep

Sleep diaries were included in the daily AM survey. As part of the daily sleep diary, participants were asked questions about their previous night’s sleep, such as “What time did you go to sleep?” (i.e., time to sleep), “How long did it take you to fall asleep (in minutes)?” (i.e., sleep latency), “How many times did you wake up, not counting your final awakening?” (i.e., frequency of nocturnal awakenings), “In total, how long did those awakenings last (in minutes)?” (i.e., wake after sleep onset), and “After your final awakening, how long did you spend in bed trying to sleep (in minutes)?” (i.e., early morning awakenings). Sleep was also quantified in two ways: total wake time (TWT) and sleep quality (SQ). TWT was equal to the sum (in minutes) of the participant’s responses to the questions about sleep latency (SL), wake after sleep onset (WASO), and early morning awakenings (EMA) [TWT = SL + WASO + EMA]. TWT encapsulates the different subtypes of insomnia—i.e., difficulty with falling asleep, staying asleep, and waking up earlier than expected. SQ was assessed by asking participants to rate on a five-point Likert scale “the quality of your sleep” from “very poor” (0) to “very good” (4).

#### 2.2.3. Physical Activity

A modified version of the International Physical Activity Questionnaire—Short Form (IPAQ) was used to assess physical activity (PA) and was given to participants as part of the daily PM survey. IPAQ has been previously validated with device-based measures [41,42]. While the IPAQ is typically used to assess physical activity during the past seven days, the questionnaire was modified to ask about physical activity in the past 24 h. Separate questions were used for vigorous, moderate, and walking activities (e.g., “During the last 24 h, how much time did you spend doing vigorous physical activities such as heavy lifting, digging, aerobics, or fast bicycling?”). Metabolic equivalent (MET) values were calculated according to standardized procedures to calculate the total MET-minutes [43]. In the analyses below, physical activity was operationalized in terms of MET minutes.

#### 2.2.4. Depressive Symptoms

A modified version of the Patient Health Questionnaire (PHQ-9) was used to assess daily depressive symptoms. The PHQ-9 is a 9-item, self-report instrument with excellent reliability and validity [44] and can be used as a screening tool for depression [45]. In the current study, the sleep and suicide items were removed. The sleep item was removed due to possible shared variance with the sleep variables listed above and the suicide item was removed due to the nature of the study (i.e., names or other identifiable information were not collected and therefore following up in the event of a high-risk situation would not have been possible). Total scores on the full 7-item scale range from 0–21. The PHQ-7 showed good internal consistency in the sample (α = 0.87). The PHQ-7 was included as part of the daily PM survey.

### 2.3. Statistical Analyses

Descriptive statistics were first computed to report the means, standard deviations, and Pearson correlations between all variables. This was followed by separate mixed-effects models (SPSS MIXED), which included days (Level 1) nested within participants (Level 2). All variables that were assessed in the evening were lagged in order to estimate the association between sleep variables (AM assessment) and social, mood, and physical activity variables (PM assessment). All main effects (for social, sleep, and physical activity variables) and two-way interactions were entered as fixed effects, and intercepts were allowed to vary randomly. The effects of social isolation and dissatisfaction on depressive symptoms (dependent variable) were assessed separately. Age, gender, race, marital status, employment status, and educational attainment were entered as covariates in the initial main effects model to control for the variance in depressive symptoms that is accounted for by these variables. The distributions for total wake time and physical activity were positively skewed, and therefore, extreme responses at the upper 2% of the distribution were winsorized (i.e., the top 2 percentile values were recorded and set at the 98th percentile value). All continuous predictor variables were mean-centered.

## 3. Results

### 3.1. Descriptive Statistics

Social isolation ratings ranged from 0–100, with a mean rating of 29.6 (SD = 29.0), whereas social satisfaction ratings ranged from 1–7, with a mean rating of 5.4 (SD = 1.4). For the sleep items, average TWT was 64.8 min (SD = 69.8; range = 0–385) and average SQ rating was 2.6 (SD = 1.0; range = 0–4). Average PA score was 308.0 (SD = 389.8; range = 0–1768). The average PHQ-7 score was 3.0 (SD = 3.9; range = 0–21). There was a moderately strong, negative correlation between social isolation and satisfaction, r = −0.46, *p* < 0.001. While SQ had a small, but significant, correlation with social isolation, r = −0.13, *p* < 0.001, and social satisfaction, r = 0.18, *p* < 0.001, the correlations with TWT and PA were less than r = 0.10. Depressive symptoms were significantly correlated with all independent variables: social isolation, r = 0.37, social satisfaction, r = −0.39, TWT, r = 0.17, SQ, r = −0.31, and PA, r = −0.11 (all significant at *p* < 0.001).

### 3.2. Regression Analyses

Results from an initial regression model tested the main effects of social isolation and social satisfaction on depressive symptoms, while controlling for age, gender, race, marital status, employment status, and educational attainment. According to the results, greater depressive symptoms were independently related to greater social isolation, b = 0.03, t(24,479) = 35.9, *p* < 0.001, and less satisfaction with one’s social interactions, b = −0.44, t(23,916) = −32.7, *p* < 0.001. Next, the main effects of TWT, SQ, and PA on depressive symptoms were assessed (while controlling for all covariates). Greater TWT, b = 0.001, t(17,404) = 3.9, *p* < 0.001, lower SQ, b = −0.24, t(17,902) = −11.4, *p* < 0.001, and lower PA, b = −0.0007, t(18,513) = −12.9, *p* < 0.001, were all significantly related to greater depressive symptoms.

### 3.3. Moderation Analyses—Social Isolation

**Figure 1:**
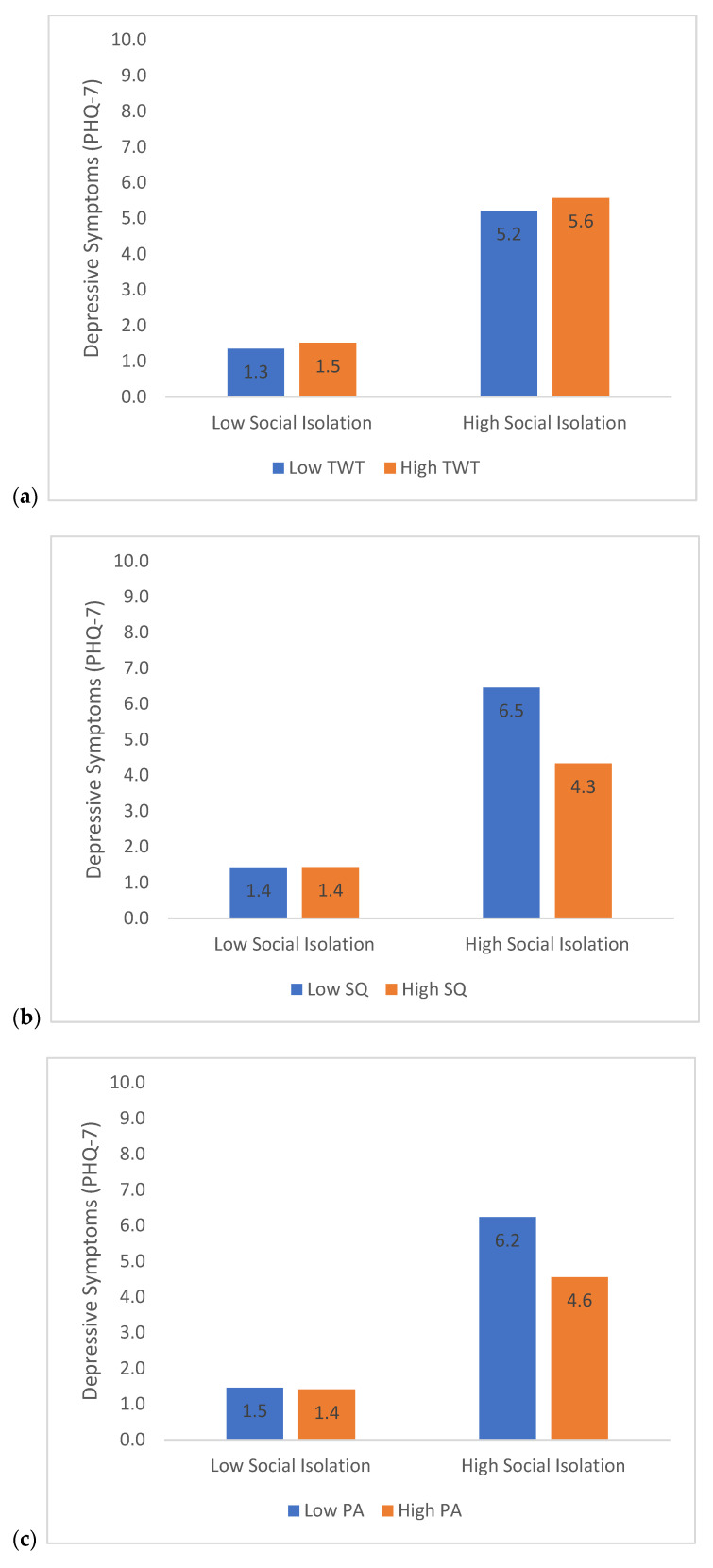
Estimated depressive symptoms (PHQ-7 scores) as a function of the association between social isolation and (**a**) total wake time (TWT), (**b**) sleep quality (SQ), and (**c**) physical activity (PA). High and low social isolation scores represent ± 2 SD from the sample mean ± 2 SD from the sample mean was also used to estimate high and low values for TWT, SQ, and PA.

Results from the first moderation test were consistent with the main effects model. Social isolation, TWT, SQ, and PA were all significantly related to depressive symptoms. Greater social isolation, b = 0.03, t(18,930) = 43.0, *p* < 0.001, greater TWT, b = 0.001, t(17,266) = 3.6, *p* < 0.001, lower SQ, b = −0.27, t(17,855) = −13.3, *p* < 0.001, and lower PA, b = −0.0005, t(18,498) = −10.3, *p* < 0.001, all predicted greater depressive symptoms in the evening. These results were qualified by significant two-way interactions between social isolation and SQ, b = −0.005, t(17,550) = −7.4, *p* < 0.001, and social isolation and PA, b = −0.00001, t(17,903) = −5.6, *p* < 0.001. Please see Table 2 for all model estimates. PHQ-7 scores are plotted in Figure 1 to illustrate the associations.

### 3.4. Moderation Analyses—Social Satisfaction

**Figure 2:**
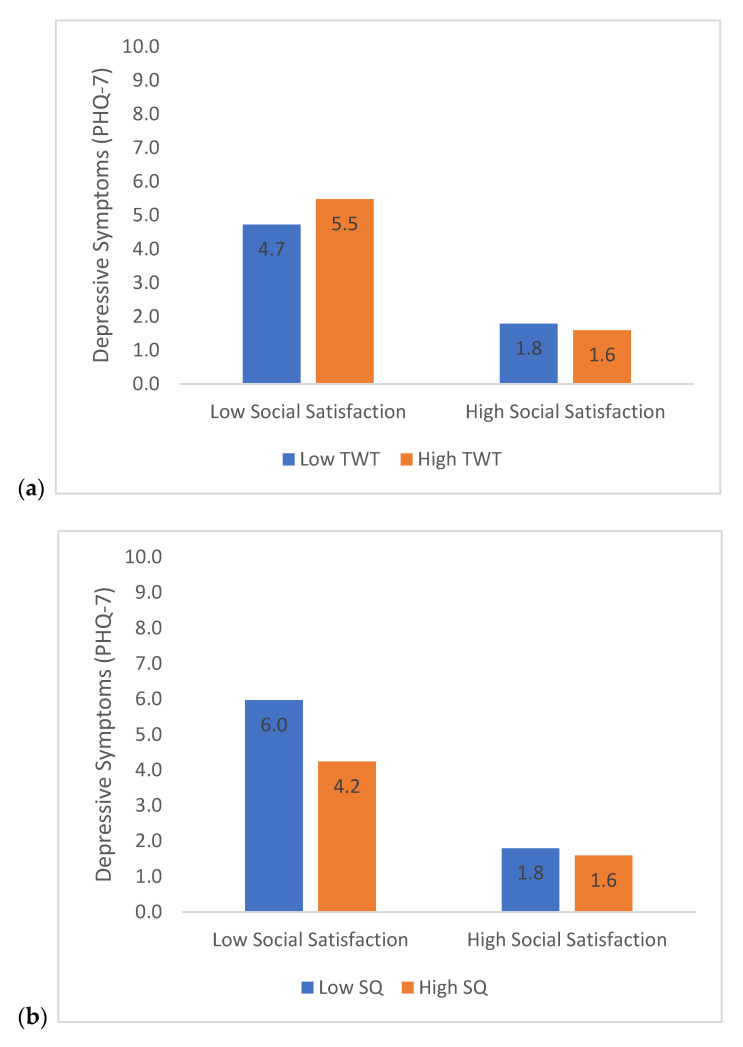

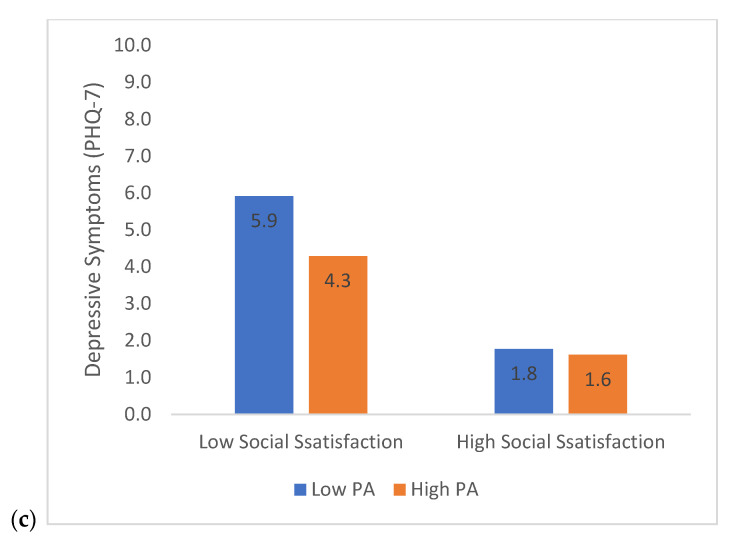
Estimated depressive symptoms (PHQ-7 scores) as a function of the association between social satisfaction and (**a**) total wake time (TWT), (**b**) sleep quality (SQ), and (**c**) physical activity (PA). High and low social satisfaction scores represent ± 2 SD from the sample mean ± 2 SD from the sample mean was also used to estimate high and low values for TWT, SQ, and PA.

Results from the second moderation test also supported that lower social satisfaction, b = −0.59, t(18,874) = −41.0, *p* < 0.001, greater TWT, b = 0.001, t(17,681) = 4.0, *p* < 0.001, lower SQ, b = −0.25, t(18,247) = −12.2, *p* < 0.001, and lower PA, b = −0.0005, t(18,886) = −10.8, *p* < 0.001, were associated with greater depressive symptoms. These effects were qualified by significant two-way interactions between social satisfaction and TWT, b = 0.0006, t(17,401) = −3.8, *p* < 0.001, social satisfaction and SQ, b = 0.068, t(17,784) = 5.5, *p* < 0.001, and social satisfaction and PA, b = 0.0002, t(18,101) = 5.3, *p* < 0.001 (Figure 2).

## 4. Discussion

As expected, social isolation and social satisfaction were each strongly related to greater depressive symptoms. That is, individuals were more likely to experience depressive symptoms in the evening if they are feeling socially isolated or dissatisfied with the quality or quantity of their social interactions that day. These results are consistent with research indicating that a lack of social connectedness or belongingness is a risk factor for greater depression [18]. For many people, the quantity or duration of face-to-face interactions has decreased dramatically since the COVID-19 pandemic. Being able to physically interact with others is an important aspect of being socially connected. COVID-19 has led to a larger reliance on virtual communication platforms. Even with these virtual interactions, people’s social needs are not being met, leading to greater feelings of social isolation or dissatisfaction, and ultimately, greater depression.

Poor sleep quality was also independently related to greater depressive symptoms and significantly modulated the impact of both social isolation and satisfaction on depression. When examined in the context of social isolation/satisfaction, the data showed that the relative difference in depressive symptoms varied as a function of sleep quality. For example, according to the model estimates (see Figure 1b and Figure 2b), for high social isolation, the difference in depressive symptoms from high to low sleep quality was 2.2, whereas for low social isolation, mean depressive symptom scores were much lower and the difference between high and low sleep quality was equal to zero. These findings suggest that while social isolation (or dissatisfaction) negatively impacts psychological health, these effects are buffered by good sleep. In contrast, the moderating effect of TWT was not as strong (only a 0.4–0.8 point difference on the PHQ scores between low and high TWT), and in the case of social isolation, not statistically significant. Overall, these results are in line with other research suggesting that poor sleep has been related to increased depression during COVID-19 [46,47]. These findings are also consistent with clinical recommendations that maintaining good sleep during social quarantine is important [48,49].

The exact mechanisms by which sleep impacts the social distancing-depression link is unknown, however, there are several possibilities. The most likely possibility is that sleep is related to the physiological and psychological processes that respond to, and help us cope with, stress (e.g., down-regulating hormonal and sympathetic responses, up-regulating executive functioning). Good sleep may subserve cognitive and/or mood regulatory functions. Lack of sleep or poor sleep quality is known to intensify a negative mood, disrupt emotion regulation, or impact executive control [50,51,52,53]. Taken together, it may be the case that without [good] sleep, we do not have the cognitive resources/bandwidth to cope with the negative thoughts and emotions that come with feeling socially isolated or dissatisfied.

Depressive symptoms were also related to differences in physical activity (PA), with lower PA predicting greater depression scores. Prior studies showed a similar link between reduced PA and greater depression symptoms during the pandemic [54,55,56]. Like sleep quality, the effect of social isolation and satisfaction on depressive symptoms varied as a function of PA. For example, for those participants who reported low levels of social satisfaction, depressive symptoms were greatest if they also reported low PA (Figure 2c). The mechanism here may be related to the notion that engaging in PA is a form of behavioral activation, and therefore, the overall impact of social isolation and dissatisfaction on mood can be attenuated by exercising. Greater PA may also impact physiological and hormonal processes that have been implicated in the regulation of mood. For example, aerobic exercise increases serotonin and dopamine concentrations and availability in the brain [57,58], thus leading to more positive emotional processing and reducing depressive symptoms [59]. PA can also regulate the release of corticotropin releasing hormone from the hypothalamus and adrenocorticotropic hormone from the pituitary gland, and subsequently, decreasing cortisol blood levels as glucocorticoid receptor sensitivity increases post-exercise [60,61]. Exercising regularly promotes healthy hypothalamic pituitary adrenal axis functioning, allowing for healthier responses to stress [62].

### Strengths and Limitations

The present study has several important strengths and limitations. It is one of the first studies to quantify the overall social impact of COVID-19 on psychological health, and more specifically, provides some of the first empirical data to support the recommendations that getting good sleep and being physically active during social quarantine is important for your mental health. It is important to highlight that social or physical distancing (e.g., to what extent are people physically distant from others) was not assessed. While relevant, social distancing is difficult to measure given that it is relative (the extent to which people are socially active under normal conditions varies considerably) and since it was not possible to collect social engagement prior to COVID-19, these data would have been more difficult to interpret. It is also worth noting that this study surveyed people from across the United States, where social restrictions and stay-at-home orders varied considerably between states during these early months of the pandemic. Moreover, the current study focused on the unintended effects of social restrictions during the early stages of the pandemic. It is important to keep in mind that there were several other factors influencing feelings of stress, depression, and anxiety during this time, such as knowledge about the virus, living situation, or financial stability [63]. The present study did not use the full-scale PHQ-9. While the PHQ-8 (no suicide item) has been validated and previously used in population-based research [64], the PHQ-7 (no sleep item) is not commonly used. Future studies should consider using other measures or methods for assessing depression. In addition, PHQ-7 scores were relatively low. This is not surprising given that a community sample was used. Whether or not these findings will generalize to clinical samples is unknown. This study was cross-sectional in nature, and therefore, causal relationships cannot be determined. It may be the case, for example, that feeling socially isolated or dissatisfied may be a consequence of being depressed and not vice versa. This concern is minimized, however, given that the present data included a mixed model approach to look at daily associations in these variables. The findings are also limited by the nature of the sample. The sample was not diverse with regard to gender or race, which limits its generalizability to other groups. Finally, the focus of this paper was on the psychological effects of social distancing, and not COVID-19 (i.e., research on those that contracted the virus). Follow-up assessments are currently ongoing to quantify whether some of these variables (poor sleep, physical activity) are related to risk for infection, as well as the severity and duration of COVID-19 symptoms.

## 5. Conclusions

The significance of the current study is that it provides empirical evidence that social distancing-related isolation and social dissatisfaction are associated with depressive symptomatology in a community sample. Moreover, good sleep and physical activity attenuated those effects. While this is only preliminary evidence in support of regular sleep and exercise during a pandemic, these findings highlight the importance of gaining a better understanding of the mechanisms by which sleep and physical activity modulate the effects of social isolation/dissatisfaction on psychological health. At the onset of COVID-19, multiple recommendations were made by scientific and public health officials for how to minimize the negative effects of social isolation (including getting good sleep and exercise). While at the time these recommendations were not based on data, we are happy to provide some data to support those initial recommendations.

## Figures and Tables

**Table 1:** Percentages and sample size for all demographic variables.

	*n*	%
**Gender**		
Female	2885	78.9
Male	691	18.9
Transgender Female	4	0.1
Transgender Male	14	0.4
Gender Variant/Non-Conforming	42	1.1
Other	19	0.5
**Race/Ethnicity**		
American Indian, Native American, or Alaska Native	18	0.5
Asian or Asian American	150	4.1
Black, African American, or African	175	4.8
Latino or Latina	117	3.2
Middle Eastern or Arab	12	0.3
Native Hawaiian or Other Pacific Islander	5	0.1
White or Caucasian	3042	83.2
Multi-racial	102	2.8
Other	33	0.9
**Marital Status**		
Married	1639	44.8
Widowed	136	3.7
Divorced	506	13.8
Separated	52	1.4
Never Married	1317	36.0
**Educational History**		
Less than high school	11	0.3
High school graduate	169	4.6
Some college	494	13.5
2-year degree	276	7.5
4-year degree	1313	35.9
Professional degree	1108	30.3
Doctorate	272	7.4
**Employment**		
Unemployed	1314	35.9
Employed 1–20 h	348	9.5
Employed 20–30 h	231	6.3
Employed full time (40+ h)	1744	47.7
**Work Shift**		
First (9 a.m.–5 p.m.)	1964	53.7
Second (4 p.m.–12 a.m.)	128	3.5
Third (12 a.m.–8 a.m.)	45	1.2
PTE (less than 3 day per week)	145	4.0
Work at home	949	25.9

**Table 2:** Unstandardized model estimates and t-values for the moderation analyses. For both models, all main effects and interaction terms were entered as fixed effects. The dependent variable was depressive symptoms (PHQ-7 scores).

	*b*	*t*	*p*-Value
**Model 1**			
Social Isolation	0.034	43.04	<0.001
Total Wake Time (TWT)	0.001	3.65	<0.001
Sleep Quality (SQ)	−0.272	−13.25	<0.001
Physical Activity (PA)	−0.001	−10.35	<0.001
Social Isolation × TWT	0.00001	0.79	0.43
Social Isolation × SQ	−0.005	−7.39	<0.001
Social Isolation × PA	−0.00001	−5.59	<0.001
**Model 2**			
Social Satisfaction	−0.591	−41.00	<0.001
Total Wake Time (TWT)	0.001	3.97	<0.001
Sleep Quality (SQ)	−0.249	−12.22	<0.001
Physical Activity (PA)	−0.001	−10.84	<0.001
Social Satisfaction × TWT	−0.001	−3.76	<0.001
Social Satisfaction × SQ	−0.068	5.54	<0.001
Social Satisfaction × PA	−0.0002	5.35	<0.001

All predictor variables were mean-centered, and TWT/PA were winsorized.

## Data Availability

Data is available from the corresponding author upon request.
